# The status of depression literacy and its relationship with quality of life among Iranian public population: a cross sectional study

**DOI:** 10.1186/s12888-022-04251-0

**Published:** 2022-09-13

**Authors:** Hadi Tehrani, Mohebat Vali, Mahbobeh Nejatian, Mahdi Moshki, Elham Charoghchian Khorasani, Alireza Jafari

**Affiliations:** 1 Social Determinants of Health Research Center, Mashhad University of Medical Sciences, Mashhad, Iran; 2 Department of Health Education and Health Promotion, School of Health, Mashhad University of Medical Sciences, Mashhad, Iran; 3 Student Research Committee, Shiraz University of Medical Sciences, Shiraz, Iran; 4 Social Determinants of Health Research Center, Gonabad University of Medical Sciences, Gonabad, Iran; 5 Department of Health Education and Health Promotion, School of Health, Social Determinants of Health Research Center, Gonabad University of Medical Sciences, Gonabad, Iran; 6 Department of Health Education and Health Promotion, School of Health, Social Development and Health Promotion Research Center, Gonabad University of Medical Sciences, Gonabad, Iran

**Keywords:** Mental health literacy, D-Lit, SF-12, Quality of life, Depression, Mental health

## Abstract

**Background:**

This study was designed and conducted to determine the status of depression literacy (D-Lit) and its relationship with the quality of life across the Iranian population.

**Methods:**

This cross-sectional study was conducted in 2020 among 1382 participating from the general population in Gonabad, Iran. Participants were selected and recruited using a multistage sampling method. Data were collected using three sets of questionnaires (demographic section, D-Lit scale, and quality of life questionnaire). Data were analyzed by SPSS software version 24 and using independent samples t- test, Chi-square test, One-way ANOVA, and Pearson correlation.

**Results:**

Based on the results, the mean (± standard deviation) of D-Lit and quality of life were 44.14 (± 4.69) and 35.81 (± 5.17), respectively. Based on the results of Pearson correlation coefficient, there was a significant positive correlation between D-Lit and quality of life (*r* = 0.104, *p* < 0.001). D-Lit was significantly higher in those who reported having consulted with a psychiatrist compared with those who did not (*p* < 0.001). Participants with a family history of mental illness and those whose family members were referred to a psychologist for psychological issues had significantly higher levels of D-Lit than others (*p* < 0.001).

**Conclusion:**

Based on the results of this study, some D-Lit projects should be improved. Also, the results revealed that D-Lit is a potential factor that can affect people's mental health status and enhance their quality of life. Therefore, it is necessary to develop appropriate educational programs to enhance D-Lit in the community and ultimately improve the quality of life in the community by reducing mental illness.

## Background

Depression literacy (D-Lit) is a special type of mental health literacy and is defined as the ability to recognize depression as well as make informed decisions about treatment [1]. The results of a study in Iran showed that the state of mental health literacy in the general population is not enough [2]. A study in Iran showed that D-Lit was low, with 48.5% of participants failing to identify mental disorders and 47.5% not intending to help search [3]. Higher D-Lit is associated with appropriate help [4], and adequate D-Lit can improve prevention, early detection, intervention, and prognosis of depression [5].

Depression is one of the top four causes of disability globally [6], and by 2030, depression will be the leading cause of the world's disease burden [7]. It is a common, chronic, recurrent, and treatable disorder associated with illness and death [8]. Depression is characterized by disturbed mood, lack of happiness, sleep problems, weight changes, feelings of guilt, inattention, daily dysfunction, and in severe cases, suicide [9–11].

Depression is associated with decreased of quality of life and can have a significant impact on the quality of life [8, 12]. The World Health Organization defines the quality of life as “an individual's perception of their position in life in the context of the culture and value systems in which they live and in relation to their goals, expectations, standards and concerns” [13]. Thus, the quality of life is a subjective and multidimensional issue and related to people's understanding of various aspects of life [14]. Quality of life can predict the extent and impact of illness, injury, disability, and assess community mental health [15]. Various studies have examined factors associated with quality of life [16–20], but these studies have not investigated various aspects of D-Lit and quality of life. Therefore, this study aimed to determine the status of D-Lit and its relationship with the quality of life of the Iranian population.

## Methods

This cross-sectional study was conducted in 2020 among 1382 participating from the general population in Gonabad, Iran (Gonabad is one of the cities in Khorasan Razavi province in eastern Iran).

### Sample size

Based on a similar study [21], the sample size of this study was considered to be 1382 participants (0.95% confidence level, D-Lit rate/ *p* = 0.53, accuracy/d = 0.03, 25% of sample loss).

### Sampling method

The multi-stage sampling method was used to select and include the participants. In the first stage, proportional stratified sampling is used, and in the second stage, a simple random sampling method is used. In the proportional stratified sampling method, all health centers (*n* = 3) with their population were identified and then each center was considered as a stratum. In the next step, the required sample size was selected from each center by simple random sampling from among those willing to participate in the study and who met the inclusion criteria for entry into the study. In the end, 553 people in the No. 1 health center, 525 people in the No. 2 health center, and 304 people in the health center. No. 3 entered the study. It should be noted that health centers are different from medical centers in Iran. The most important purpose of these centers is to provide primary health care services to all target populations, usually to people of all age groups.

To collect the data, the questioners referred to the health center, and after explaining the purpose of the study to the participants, the questionnaire was provided and completed by self-report. Also, questionnaires for illiterate participants were completed by questioners who were not part of the research team. In this study, inclusion criteria were e.g. participants residing in Gonabad, age 18 years and older, no specific medical conditions, and signed informed consent.

### Data Collection Instruments

Data were collected using three sets of questionnaires, including a demographic questionnaire, a D-Lit questionnaire, and a quality of life questionnaire (SF-12).

- *1) Demographic questionnaire:* In this section, data were collected on sex, job status, marital status, age group, income, education level, participants consulted psychologist / psychiatrist for psychological issues, family history of mental illness, obtain information related to mental illness, and refer your family member to a psychologist/psychiatrist for psychological issues.
- *2) D-Lit Questionnaire:* This questionnaire was designed and confirmed by Griffiths et al. and includes 22 items that survey the status of D-Lit [22, 23]. These items are assessed on a three-point scale (true, false, I don't know) with a score range of 22 to 66. For each correct answer, a score is assigned, with higher scores indicating better D-Lit status. Based on the results of Griffiths study, Cronbach's alpha and 3 month test–retest were reported 0.70 and 0.71, respectively [22]. The validity and reliability of the Persian version of the D-Lit questionnaire was investigated in the Tehrani study [24]. Based on exploratory factor analysis, 5 factors with eigenvalues greater than 1 were extracted, accounting for 56.30% of the variance (F1: Knowledge of the psychological symptoms, F2: Knowledge about the effectiveness of available treatment methods, F3: Knowledge about cognitive-behavioral symptoms, F4: Knowledge about taking medications and their side effects, and F5: Knowledge of severity of disease) [24]. In the confirmatory factor analysis, these five factors were examined, 1 question was removed and the final questionnaire was confirmed with 21 items with a score range of 21 to 63. Finally, Cronbach's alpha coefficient for the all items was 0.890 [24].
- *3) Quality of life questionnaire (SF-12):* This tool was designed by Ware and Keller [25]. This questionnaire is a shortened version of quality of life with 8 subscales and contains 12 items [25]. This instrument examines the quality of life in terms of role limitations due to physical problems (RP-2 questions), bodily pain (BP-1 question), physical functioning (PF-2 questions), role limitations due to emotional problems (RE-2 questions), general health (GH-1 questions), social functioning (SF-1 question), vitality (VT-1 question), and perceived mental health (MH-2 questions). These 8 variables are divided into two subscales of Physical Health with 6 items (RF, RP, BP, GH), and Mental Health with 6 items (SF, RE, VT, MH). The validity and reliability of the Persian version of this questionnaire was confirmed by Montazeri et al. and Cronbach's alpha for subscales of Physical Health and Mental Health were 0.73 and 0.72, respectively [26].

### Statistical analysis

This study was performed using software version 22. Independent samples t-test, Chi-square test, One-way ANOVA, and Pearson correlation test have been used for data analysis. In this study, for data analysis, a significance level of less than 0.05 was considered.

## Results

In this study, the majority of participants were female (*n* = 781, 56.9%), 79.1% (*n* = 1009,) lived in urban, 67.6% (*n* = 922) were in the age group of 18 to 35 years, and 69.1% (*n* = 942) were married. The majority of them (*n* = 858, 64.8%) had an academic level of education and 65.9% of them (*n* = 850) had medium income. In terms of job status, 33.9% (*n* = 444) were laborers and 30% (*n* = 393) were employed. In this study, only 15.5% (*n* = 210) of participants stated that they had referred to a psychologist for psychological issues, 14.5% (*n* = 198) reported that there was a history of mental illness among their family members, and 18.4% (*n* = 245) also stated that some of their family members had a history of referred to a psychologist. The majority of participants (*n* = 1061, 77.3%) reported that they received information about mental disorders.

According to the results of Table 1, there was a significant relationship between sex and D-Lit and women had higher level of D-Lit (*p* < 0.001). Also, 63.7% of women answered most of the questions correctly (*p* < 0.001). The results showed a significant relationship between age groups and D-Lit levels (*p* < 0.001). The level of education of the study participants was significantly related to their level of D-Lit, and people with a higher education level had a higher level of D-Lit (*p* < 0.001). Also, 65% of participants who had an academic level were able to answer most of the questions correctly (*p* < 0.001) (Table 1).

**Table 1:** Frequency distribution of demographic factors (*n* = 1382)

**Variables**		**n (%)**	**Depression literacy status** **n (%)**				***P*-value **^**c**^	**D- Lit**	***P*-value**
			**Incorrect response /Don’t know**	**Correct response to 1–7 questions**	**Correct response to 8–14 questions**	**Correct response to 15 questions and more**		**Mean (SD)**	
**Sex**	Female	781 (56.9)	16 (2)	267 (34.2)	461 (59)	37 (4.7)	< 0.001	44.68 (4.70)	< 0.001^a^
	Male	591 (43.1)	25 (4.2)	251 (42.5)	298 (50.4)	17 (2.9)		43.46 (4.56)	
**Age group**	18–35	922 (67.6)	28 (3)	337 (36.6)	518 (56.2)	39 (4.2)	0.599	44.52 (4.61)	< 0.001^b^
	36–50	392 (28.7)	9 (2.3)	159 (40.6)	209 (53.3)	15 (3.8)		43.49 (4.87)	
	> 50	50 (3.7)	2 (4)	20 (40)	28 (56)	0		42.60 (3.63)	
**Occupation statues**	Housewife	227 (17.3)	6 (2.6)	95 (41.9)	122 (53.7)	4 (1.8)	0.028	43.77 (4.68)	0.260^b^
	Employed	393 (30)	5 (1.3)	132 (33.6)	233 (59.3)	23 (5.9)		44.43 (4.66)	
	Self-employed	245 (18.7)	11 (4.5)	100 (40.8)	127 (51.8)	7 (2.9)		43.95 (4.43)	
	Laborer	444 (33.9)	15 (3.4)	168 (37.8)	244 (55)	17 (3.8)		44.34 (4.77)	
**Marital status**	Married	942 (69.1)	26 (2.8)	352 (37.4)	526 (55.8)	38 (4)	0.813	44.02 (4.67)	0.163^a^
	Single	422 (30.9)	15 (3.6)	163 (38.6)	228 (54)	16 (3.8)		44.41 (4.77)	
**Education level**	Illiteracy	10 (0.7)	0	4 (40)	6 (60)	0	< 0.001	44.30 (2.83)	< 0.001^b^
	Elementary	33 (2.5)	1 (3)	12 (36.4)	20 (60.6)	0		42.87 (3.14)	
	High school	423 (31.9)	19 (4.5)	196 (46.3)	206 (48.7)	2 (0.5)		42.84 (4.46)	
	Academic	858 (64.8)	18 (2.1)	282 (32.9)	508 (59.2)	50 (5.8)		44.87 (4.72)	
**Financial status**	Good	298 (23.1)	6 (2)	100 (33.6)	174 (58.4)	18 (6)	0.034	44.71 (5.06)	0.080^b^
	Medium	850 (65.9)	21 (2.5)	328 (38.6)	472 (55.5)	29 (3.4)		44.05 (4.53)	
	Weak	141 (10.9)	9 (6.4)	54 (38.3)	72 (51.1)	6 (4.3)		43.87 (4.75)	
**Participants consulted psychologist / psychiatrist for psychological issues**	Yes	210 (15.5)	1 (0.5)	50 (23.8)	142 (67.6)	17 (8.1)	< 0.001	45.74 (5.03)	< 0.001^a^
	No	1144 (84.5)	39 (3.4)	458 (40)	610 (53.3)	37 (3.2)		43.88 (4.56)	
**Family history of mental illness**	Yes	198 (14.5)	1 (0.5)	51 (25.8)	133 (67.2)	13 (6.6)	< 0.001	45.63 (4.74)	< 0.001^a^
	No	1168 (85.5)	40 (3.4)	468 (40.1)	619 (53)	41 (3.5)		43.87 (4.65)	
**Refer your family members to a psychologist / psychiatrist for psychological issues**	Yes	245 (18.4)	0	59 (24.1)	169 (69)	17 (6.9)	< 0.001	45.68 (4.78)	< 0.001^b^
	No	923 (69.5)	35 (3.8)	377 (40.8)	481 (52.1)	30(3.3)		43.85 (4.61)	
	I don’t know	160 (12)	4 (2.6)	66 (41.3)	86 (53.8)	4 (2.5)		43.50 (4.43)	
**Obtain information related to mental illness**	Yes	1061 (77.3)	25 (2.4)	372 (35.1)	613 (57.8)	51 (4.8)	< 0.001	44.48 (4.77)	< 0.001^a^
	No	311 (22.7)	16 (5.1)	145 (46.6)	147 (47.3)	3 (1)		43 (4.19)	

^a^ Independent Samples t Test

^b^ One-way ANOVA

^c^ Chi-square

The participants who reported having consulted with psychologist for their psychological issues had significantly higher levels of D-Lit compared with those who did not (*p* < 0.001). Participants with a family history of mental illness and those whose family members were referred to a psychologist for psychological issues had significantly higher levels of D-Lit than others (*p* < 0.001). Also, the participants who obtained information related to mental disorders showed significantly higher levels of D-Lit than those who did not (*p* < 0.001). There was no significant relationship between financial status and D-Lit, but people with better financial status had higher D-Lit scores (*p* > 0.05) (Table 1).

The results of Table 1 showed the status of participants' answers to the D-Lit questionnaire. Based on the results, 37.8% (*n* = 522) were able to answer 1 to 7 questions correctly, 55.4% (*n* = 765) were able to answer 8 to 14 questions correctly, and only 3.9% (*n* = 54) were able to answer 15 and more questions correctly. Of the participants, only 3% (*n* = 41) of the participants did not have D-Lit and answered all the questions incorrectly. The results of this study showed that the majority of people who answered the D-Lit questions incorrectly were male (*n* = 25), laborer (*n* = 15), married (*n* = 26), aged 18–35 (*n* = 28), had high school education (*n* = 19), and had moderate financial status (*n* = 21). More information can be seen in Table 1.

In this study, 23.7% (*n* = 327) answered all questions on knowledge of psychological symptoms subscale, 0.4% (*n* = 5) answered all questions on knowledge about the available effectiveness of treatment methods subscale, 3.1% (*n* = 43) answered all questions on knowledge about cognitive-behavioral symptoms subscale, 5.2% (*n* = 71) answered all questions on knowledge about taking medications and their side effects subscale, and 15.5% (*n* = 214) answered all questions on knowledge of severity of disease subscale (Table 2).

**Table 2:** Participants' response to the D- Lit questionnaire

**Subscale**	**Items**	**n (%)**		
		**Correct response**	**Incorrect response**	**Don’t know**
**F1: Knowledge of the psychological symptoms**	1. People with depression may feel guilty when they are innocent. (True)	868 (63)	235 (17.1)	275 (20)
	2. Loss of confidence and low self-esteem may be a sign of depression. (True)	779 (57.4)	287 (21.2)	290 (21.4)
	3. Too little or too much sleep can be a symptom of depression. (True)	867 (63.6)	216 (15.8)	280 (20.5)
	4. Eating too much or losing interest in food may be a symptom of depression. (True)	699 (51)	332 (24.2)	339 (24.7)
	5. People may move more slowly or become agitated due to their depression. (True)	782 (57.3)	220 (16.1)	363 (26.6)
**F2: Knowledge about the effectiveness of available treatment methods**	6. Clinical psychologists can prescribe antidepressant medications. (False)	369 (27.1)	454 (33.3)	540 (39.6)
	7. Many treatments for depression are more effective than antidepressant medications. (False)	632 (46.1)	224 (16.4)	514 (37.5)
	8. The effects of counseling are similar to those of cognitive- behavioral therapies for depression. (False)	695 (50.6)	215 (15.6)	464 (33.8)
	9. The effect of cognitive-behavioral therapies is the same as that of antidepressant medications for mild to moderate depression. (True)	458 (33.3)	243 (17.7)	673 (49)
**F3: Knowledge about cognitive-behavioral symptoms**	10. People with depression often speak sporadically and irrelevantly. (False)	550 (40)	419(30.5)	407 (29.6)
	11. Reckless and foolhardy behavior is a common symptom of depression. (False)	392 (28.6)	485 (35.4)	492 (35.9)
	12. Not walking on cracked and broken sidewalks may be a symptom of depression. (False)	235 (17.1)	641 (46.8)	495 (36.1)
	13. People with depression often hear sounds that are not normally heard. (False)	443 (32.5)	390 (28.7)	528 (38.8)
	14. Depression does not affect your memory and concentration. (False)	287 (20.9)	762 (55.6)	321 (23.4)
	15. Having several distinct personalities can be a symptom of depression. (False)	514 (37.8)	388 (28.6)	456 (33.6)
**F4: Knowledge about taking medications and their side effects**	16. f all the alternative and lifestyle therapies for depression, vitamins are the most beneficial. (False)	391 (28.7)	212 (15.6)	759 (55.7)
	17. People with depression should stop taking antidepressant medications as soon as they feel better. (False)	182 (13.4)	809 (59.6)	366 (27)
	18. Antidepressant medications are addictive. (False)	505 (37.1)	373 (27.4)	484 (35.5)
	19. Antidepressant medications are usually rapid-acting. (False)	382 (28.1)	389 (28.6)	588 (43.3)
**F5: Knowledge of severity of disease**	20. Most people with depression need to be hospitalized. (False)	200 (14.6)	823 (60.2)	344 (25.2)
	21. Many celebrities have suffered from depression. (True)	347 (25.5)	439 (32.3)	573 (42.2)

Based on the results of Table 3, the mean (± SD) of D-Lit and quality of life were 44.14 (± 4.69) and 35.81 (± 5.17), respectively. The mean (± SD) D-Lit subscales and quality of life are shown in Table 3. Based on the results of Pearson correlation coefficient, there was a significant positive correlation between D-Lit and quality of life (*r* = 0.104, *p* < 0.001) (Table 4).

**Table 3:** Descriptive statistics of the D-Lit questionnaire subscale scores among general population

**Subscales**		**Item**	**Range**	**Mean**	**Standard deviation**
**D- Lit**	**Knowledge of the psychological symptoms**	5	5–15	11.84	2.70
	**Knowledge about the available effectiveness of treatment methods**	4	4–12	7.51	1.45
	**Knowledge about cognitive-behavioral symptoms**	6	6–18	12.35	2.63
	**Knowledge about taking medications and their side effects**	4	4–12	8.20	1.80
	**Knowledge of severity of disease**	2	2–6	0.85	0.66
	**Total Score of D- Lit**	21	21–63	44.14	4.69
**Quality of life**	**Physical health**	6	6–20	16.27	1.97
	**Mental health**	6	6–27	19.57	4.17
	**Total score of quality of life**	12	12–47	35.81	5.17

**Table 4:** Pearson correlation between attributes of D- Lit and quality of life

**Variables**	**1**	**2**	**3**	**4**	**5**	**6**	**7**	**8**
**1. Knowledge of the psychological symptoms**	1							
**2. Knowledge about the available effectiveness of treatment methods**	-0.141*	1						
**3. Knowledge about cognitive-behavioral symptoms**	-0.175*	0.142*	1					
**4. Knowledge about taking medications and their side effects**	-0.002	0.151*	0.124*	1				
**5. Knowledge of severity of disease**	0.162*	-0.049	0.012	0.052	1			
**6. Total Depression literacy**	0.481*	0.359*	0.541*	0.515	0.295*	1		
**7. Physical health subscale**	0.077*	-0.032	0.032	0.004	-0.045	0.059*	1	
**8. Mental health subscale**	0.095*	0.012	0.004	0.088*	-0.059*	0.099*	0.283*	1
**9. Quality of Life**	0.108*	-0.012	0.019	0.073*	-0.066*	0.104*	0.623*	0.929*

^*^P < 0.001

## Discussion

This study aimed to determine the status of D-Lit and its relationship with the quality of life of the Iranian population. According to the results of this study, most of the participants did not have an adequate level of D-Lit and only a small number had an adequate level of D-Lit. The results of this study was similar with other studies [11, 27, 28]. The results of Bernstein's study showed that participants answered correctly 41.73% of the questions [29]. Results from another study showed that participants answered 52.8% of the questions [21].

The results of this study also showed that there was a significant positive correlation between D-Lit and participants' quality of life. With the development of medical technology and the improvement of life expectancy, people are paying more and more attention to their quality of life. Health managers and researchers are more concerned about whether improving D-Lit improves people's quality of life [30]. We found in our study that people with low of D-Lit had a low quality of life. This suggests that people with low D-Lit may be less concerned about their health and thus have unhealthy behaviors that reduce their quality of life. Evidence supports a significant longitudinal relationship between literacy skills and depressive symptoms and that people with low health literacy are three times more likely to suffer from depression [31]. Health skills and D-Lit refer to an individual's ability to translate mental health knowledge into healthy behaviors.

Based on the results of this study, there was a significant relationship between sex and D-Lit, with women having significantly higher levels of D-Lit than men. The results of various studies have also indicated that women have a higher level of D-Lit than men [32, 33], are more able to diagnose mental disorders [34, 35], and are more likely than men to seek occupational mental health services [36]. In addition, women tend to learn more about mental health and are more likely to interact with people with mental disorders [37, 38]. This sex difference may be related to the lower prevalence of depression in men and thus their lack of exposure to disorder. However, this lack of awareness may also cause fewer men seeking depression. Men also attribute depression primarily to weak personality and believe in the use of alcohol for relaxation [39, 40]. Men may need to be targeted as a way to improve their mental health literacy for increasing the proper amount of support.

The findings of this study also showed a significant relationship between age groups and D-Lit levels, with D-Lit rates higher in the 18- to 35-year-old age group than in other age groups. These findings are consistent with previous studies showing that older populations have poorer D-Lit [41, 42]. Poor D-Lit among older adults may be due to a variety of reasons. Mental health information may be provided through media that are less commonly used by older adults, such as the Internet. School curricula are a major source of mental health literacy for young people, but such programs have not been systematically available in recent decades. Current mental health literacy campaigns may not target the elderly. Social stigma may also be higher in older adults and prevent them from receiving information about mental health literacy [43].

Based on the results of this study, there was a significant relationship between participants' education level and their D-Lit and with increasing education level, D-Lit level also increases. Previous research has shown that people with higher education are more likely to be diagnosed with depression than people with less education [44]. At the same time, according to previous research, with the improvement of education level, the quality of life will be significantly improved in both physical and psychological dimensions. [45]. Education and area of residence appear to be significant predictors of a depression diagnosis, and people living in cities also had more Knowledge of depression [46]. Today, many villages have access to mass media (television, radio, and the Internet). Therefore, the media must provide programming in local languages to raise public awareness of mental health issues and reduce false beliefs and negative attitudes towards mental disorders.

In this study, those who reported seeing a psychologist for their psychological issues had significantly D-Lit levels than those who didn't. Also, participants who mentioned that had a family history of mental illness, and those whose family members were referred to a psychologist for psychological issues, showed significantly higher levels of D-Lit than others. Based on the results of this study, people who received information about mental disorders from various sources showed significantly higher levels of D-Lit than others. It is important to note that people often obtain mental health information from a variety of sources, which may not be reliable.; Therefore, it should be emphasized that psychologists, psychiatrists, and healthcare providers are the best sources of mental health information from which individuals should obtain reliable information [47].

People prefer to seek help from less formal sources such as the family for mental health problems [48]. Studies have shown that the most common source of help for people about health treatment is talking to family or friends [49, 50]. Seeking appropriate help from professional sources is very important for the prevention, early diagnosis, and treatment as well as recovery of mental disorders, and timely referral to these sources is essential [50, 51]. Asking less for help from a psychiatrist or psychologist may indicate poor knowledge of psychiatric services or a lack of understanding of the effective treatments offered by such specialists. Also, in another study, although many participants rated the services of psychiatrists and counselors as likely to be helpful, less than 10% of the population endorsed psychiatrists as the best help for a person with depression [40]. General practitioners or family physicians topped the list, indicating the fact that most people with depression are often seen and treated by general practitioners [40]. Behaviors of people and patients may also be influenced by culture and religion, which play an important role in mental health literacy and D-Lit [46]. In response to cultural factors, individuals may be reluctant to raise their mental health concerns with mental health professionals and psychologists. Therefore, they may decide to trust their family or friends. Also, the social stigma that manifests itself in prejudice, mistrust, stereotyping, fear, embarrassment, anger, and avoidance [52] may be another deterrent and an obstacle to utilizing services in Asian societies.

This study had some strengths and limitations. This has been a population-based study, and the use of a standard and valid assessment tool (D-Lit) has minimized some measurement bias for exposure and outcome variables. Also, this study had a good sample size. Some possible limitations have also been identified in this study. This cross-sectional study can be considered as an appropriate blueprint for discovering potential risk factors for a disease. However, the evidence presented in this study can only showed an association and is not sufficient to show a temporal relationship between exposure and outcome. This study can be considered as an exploratory study to identify a potential link between D-Lit and quality of life. Meanwhile, some important risk factors for depression, such as recent life stressors and family problems, have not been included in the analysis as potential distortions, and this may lead to biased estimation of the strength of the relationship between exposure and outcome variables. Future studies can be conducted with a better design, such as a longitudinal cohort study, and include potentially important confounding factors to determine the cause of the relationship. Also, since the questionnaires for illiterate participants were filled out by the questioners, there may be some errors in the information collected.

## Conclusion

The purpose of this study was to investigate the relationship between D-Lit and quality of life. The results revealed that D-Lit is a potential factor that can affect people's mental health status and enhance their quality of life. Increasing the level of D-Lit should be considered as an important preventive strategy for the mental problems of the community. Also, it is necessary to develop appropriate educational programs to enhance D-Lit in the community and ultimately improve the quality of life in the community by reducing mental illness. Mental health education can be provided in patient-physician communication, in training campaigns and programs such as first aid mental health skills training. Dissemination of information about evidence-based therapies should be considered in clinical practice, as well as in promotion and training programs. Also, further research on self-help for depression is needed. Such efforts should focus not only on diagnosing symptoms but also on disseminating information on risk factors, evidence-based therapies on risk factors, and depression factors.

## Data Availability

All data generated or analysed during this study are included in this published article.

## Abbreviations

D-Lit: Depression literacy
SF-12: The 12-item Short Form Health Survey
RP: Role limitations due to physical problems
PF: Physical Functioning
GH: General Health
BP: Bodily Pain
SF: Social Functioning
VT: Vitality; MH: Mental Health
RE: Role limitations due to emotional problems
